# Machine learning for effectively avoiding overfitting is a crucial strategy for the genetic prediction of polygenic psychiatric phenotypes

**DOI:** 10.1038/s41398-020-00957-5

**Published:** 2020-08-17

**Authors:** Yuta Takahashi, Masao Ueki, Gen Tamiya, Soichi Ogishima, Kengo Kinoshita, Atsushi Hozawa, Naoko Minegishi, Fuji Nagami, Kentaro Fukumoto, Kotaro Otsuka, Kozo Tanno, Kiyomi Sakata, Atsushi Shimizu, Makoto Sasaki, Kenji Sobue, Shigeo Kure, Masayuki Yamamoto, Hiroaki Tomita

**Affiliations:** 1 Department of Psychiatry, Graduate School of Medicine, Tohoku University, Miyagi, Japan; 2 Tohoku Medical Megabank Organization, Tohoku University, Miyagi, Japan; 3 Department of Disaster Psychiatry, International Research Institute of Disaster Science, Tohoku University, Miyagi, Japan; 4 RIKEN Center for Advanced Intelligence Project, Tokyo, Japan; 5 Graduate School of Information Sciences, Tohoku University, Sendai, Japan; 6 Iwate Tohoku Medical Megabank Organization, Disaster Reconstruction Center, Iwate Medical University, Iwate, Japan; 7 Department of Neuropsychiatry, Iwate Medical University, Iwate, Japan; 8 Department of Medical Biochemistry, Graduate School of Medicine, Tohoku University, Miyagi, Japan

**Keywords:** Personalized medicine, Clinical genetics

## Abstract

The accuracy of previous genetic studies in predicting polygenic psychiatric phenotypes has been limited mainly due to the limited power in distinguishing truly susceptible variants from null variants and the resulting overfitting. A novel prediction algorithm, Smooth-Threshold Multivariate Genetic Prediction (STMGP), was applied to improve the genome-based prediction of psychiatric phenotypes by decreasing overfitting through selecting variants and building a penalized regression model. Prediction models were trained using a cohort of 3685 subjects in Miyagi prefecture and validated with an independently recruited cohort of 3048 subjects in Iwate prefecture in Japan. Genotyping was performed using HumanOmniExpressExome BeadChip Arrays. We used the target phenotype of depressive symptoms and simulated phenotypes with varying complexity and various effect-size distributions of risk alleles. The prediction accuracy and the degree of overfitting of STMGP were compared with those of state-of-the-art models (polygenic risk scores, genomic best linear-unbiased prediction, summary-data-based best linear-unbiased prediction, BayesR, and ridge regression). In the prediction of depressive symptoms, compared with the other models, STMGP showed the highest prediction accuracy with the lowest degree of overfitting, although there was no significant difference in prediction accuracy. Simulation studies suggested that STMGP has a better prediction accuracy for moderately polygenic phenotypes. Our investigations suggest the potential usefulness of STMGP for predicting polygenic psychiatric conditions while avoiding overfitting.

## Introduction

**Fig. 1:**
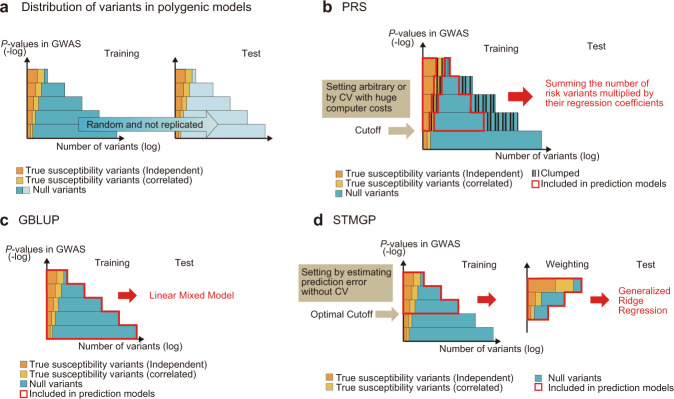
The concept of genetic architecture and predictive models for polygenic diseases. **a** The distribution of *P* values in GWAS for polygenic disease models in training and test datasets. To depict the concept of genetic architecture and predictive models for polygenic disease, the simulated distribution of variants analyzed in GWAS for a certain target phenotype is shown in the figures. The *Y* axis indicates the negative logarithm (−log) of *P* values, and the *X* axis indicates the logarithm (log) of the number of variants. While the *P* values of variants with true susceptibility to the disease of interest (depicted in orange and yellow) tend to be small, some of them can be large due to insufficient power. Likewise, while the majority of the *P* values of null variants (variants with no effect on the susceptibility to the disease, depicted in blue) tend to be large, some of them can be small by random chance due to a large number of statistical tests. The variants with true susceptibility to the disease can be divided into a set of variants that are independent of each other (depicted in orange) and a set of remaining variants that are dependent on the former variants due to the linkage disequilibrium (depicted in yellow). While true susceptibility variants increase prediction accuracy, null variants decrease prediction accuracy when the variants are included in the prediction model because associations between the null variants and the target phenotype are not replicated in the validation cohort, which is referred to as overfitting. Distinguishing true susceptibility variants and null variants in single GWAS is difficult with currently available sample-size data. **b** Concepts of PRS. PRS intends to select variants with true susceptibility and avoid influence from null variants by setting a cutoff of *P* values in GWAS; however, the model decreases prediction accuracy because the model (i) still includes and overestimates a large number of the null variants, and (ii) incorporates clumping and excludes correlated true susceptibility variants, which can contribute to prediction accuracy. **c** Concepts of GBLUP. GBLUP utilizes true susceptibility variants correlated with each other for better prediction accuracy; however, the model includes a large number of null variants and results in decreasing prediction accuracy due to overfitting. **d** Concepts of STMGP. STMGP decreases overfitting by weighting selected variants to decrease overestimation of null variants, utilizes correlated true susceptibility variants effectively by building generalized ridge regression, and sets an optimal cutoff for the *P* value with low computer costs by avoiding CV. GWAS genome-wide association study, PRS polygenic risk score, CV cross-validation, GBLUP genomic best linear-unbiased prediction, STMGP Smooth-Threshold Multivariate Genetic Prediction.

Recent genome-wide association studies (GWAS) revealed that the genetic influences on many psychiatric conditions are based on the aggregation of a large number of small effects, which is referred to as a polygenic model^1–3^. In a polygenic model, building high-performance prediction models based on GWAS in training data is challenging because selecting only truly (weakly) associated variants based on a single GWAS with the currently available largest sample size is difficult due to limited statistical power (Fig. 1a)^4,5^. The limited statistical power could cause overfitting in the genetic prediction models, which is characterized by apparent high prediction accuracies when it is calculated using the training dataset and low prediction accuracies when it is calculated using independent test datasets. One of the main reasons for overfitting is the inclusion of a large number of variants with no effect on the target disease in the prediction models. In this paper, these variants that do not influence the target phenotype will be referred to as null variants in accordance with the previous article^6^. When the statistical power was limited, the variants with *P* values lower than the genome-wide significance level include both null variants and true susceptibility variants, and Dudbridge et al.^6^ showed that the inclusion of too many null variants by setting the *P*-value cutoff of the prediction model too high would decrease the prediction accuracy calculated in independent test datasets.

There have been two frequently utilized prediction methods for complex diseases following a polygenic model: polygenic risk scores (PRS) and genomic best linear-unbiased prediction (GBLUP). PRS, proposed by Purcell et al.^7^, is calculated as a sum of the number of trait-associated alleles, defined based on an arbitrary *P*-value cutoff multiplied by their regression coefficients (Fig. 1b). Although PRS has been frequently utilized for the prediction of polygenic diseases, the prediction accuracies have been low^2,6,8,9^. In the study of genetics related to polygenic psychiatric condition, the low prediction accuracy of PRS was mainly due to limited statistical power^2,3,10^. Another limitation of PRS is the utilization of only independent SNP datasets following a clumping procedure, and the information of a group of SNPs in linkage disequilibrium (LD) is limitedly considered for prediction. Although limitations of statistical power need to be solved by a larger sample size, the effective utilization of the SNP group in LDs could be considered by building a multiple-regression model. In addition, the overfitting from null variants of PRS could be decreased to some extent by building a penalized regression model.

Another frequently utilized genomic prediction method for complex traits is GBLUP, which fits all the variants simultaneously by building linear mixed models, treating the effects of the variants as random effects (Fig. 1c)^11–13^. Unlike PRS, GBLUP can utilize a group of variants correlated with each other effectively for prediction. However, the prediction accuracy of GBLUP is also low^14^. One of the limitations of GBLUP models is that the method does not select specific variants, and includes a large number of null variants, which could decrease prediction accuracies through overfitting, as observed in PRS.

To overcome the above-mentioned problems, Smooth-Threshold Multivariate Genetic Prediction (STMGP) was proposed by Ueki and Tamiya^15^. Similar to PRS, STMGP also builds prediction models based on variants selected by the threshold *P* value of GWAS. However, STMGP overcomes the problems of PRS in the following two aspects. First, STMGP can avoid overfitting by weighting variants by the strength of marginal association reflecting the certainty of inclusion, which increases and stabilizes prediction accuracies^16^. Second, in STMGP, all the selected variants are utilized as predictor variables to build a penalized regression model, generalized ridge regression, which enables the effective utilization of correlated susceptibility variants for better prediction accuracy (Fig. 1d). The STMGP algorithm shares similarity with Elastic net, a penalized regression machine learning. Elastic net, Lasso, and other shrinkage machine-learning methods were reported to have high prediction accuracy^17,18^, but they require huge computer costs due to cross-validation for setting tuning parameters, and cannot be applied to large-scale genome-wide data^19^. In contrast, STMGP does not utilize cross-validation by estimating prediction error utilizing an unbiased Cp-type model selection criterion, and can be utilized for large-scale genome-wide data with lower computational costs.

Considering the performance of STMGP previously reported by Ueki and Tamiya^15^, we hypothesized that STMGP would have good performance in predicting polygenic psychiatric phenotypes. In this study, we first evaluated the performance of STMGP in predicting depressive symptoms based on real GWAS data, including 3685 training and 3048 validation cohorts. Depressive symptoms are one of the most polygenic phenotypes according to the previous GWAS^2,3,20^, whose SNP-based heritability has been estimated to be 0.04 (SE 0.01)^21^. Then, we also evaluated the performance of STMGP using simulated phenotypes with varying degrees of complexity and various effect-size distributions. The performance of STMGP was evaluated in terms of prediction accuracy and the degree of overfitting, and compared with that of other state-of-the-art methods, which included, in addition to PRS and GBLUP, summary-data-based best linear-unbiased prediction (SBLUP)^22^, BayesR (a Bayesian hierarchical model for complex trait analysis)^23^, and ridge regression (penalized regression model) using clumped SNP data.

## Materials and methods

### Study population, genotyping, and quality control

The genome-wide SNP data for a total of 9966 subjects, including 4974 training cohort subjects living in Miyagi prefecture recruited by Tohoku University and 4992 validation cohort subjects living in Iwate prefecture recruited by Iwate Medical University, were available at the time of the current analysis^24,25^. Both cohorts were collected in an effort to survey the health condition of residents in the prefectures affected by the 2011 Great East Japan Earthquake and Tsunami. HumanOmniExpressExome BeadChip Array (Illumina Inc., San Diego, CA, USA) was utilized for genotyping for both cohorts. Subjects with a low call rate (<0.98, *n* = 2 in the training cohort and *n* = 3 in the validation cohort) were excluded. We detected 2156 close-relationship pairs (620 in the training cohort and 1536 in the validation cohort) using the identity-by-descent method in PLINK software (PI_HAT > 0.09375)^26^ among the training cohort, the validation cohort, or between these cohorts. Then, in each of these pairs, a subject with lower call rates was excluded. Variants with low call rates (<0.99), low Hardy–Weinberg equilibrium exact-test *P* values (<1 × 10^−4^), or low minor-allele frequencies (<0.01) were filtered out. Subjects without outcome or covariate information (*n* = 669 in the training cohort and *n* = 408 in the validation cohort) were excluded. Finally, 3685 subjects in the training cohort and 3048 subjects in the validation cohort with 615,386 variants were subjected to prediction analyses. The imputed genome datasets were used in the additional analyses, and the imputation method is shown in the Supplementary Methods. All protocols of our studies were approved by the Ethics Committees of Tohoku University and Iwate Medical University. Written informed consent was obtained from all subjects. This study was carried out according to the principles expressed in the Declaration of Helsinki.

### Outcome measures (depressive symptoms)

Depressive state was evaluated by the Center for Epidemiological Studies-Depression (CES-D) score^27^. The CES-D contains 20 items, each of which is rated on a 4-point scale ranging from 0 to 3 based on the frequency of feelings and behaviors over the past 7 days, with a higher score suggesting a severe depressive state. There were 2.0% and 0.7% missing data in CES-D items in the training and validation cohorts, respectively, and listwise deletion was performed for missing data.

The distributions of CES-D scores in the current datasets are shown in Supplementary Fig. 1. Since the distribution of the raw CES-D scores was different from a normal distribution, we evaluated the influence of phenotype distributions and outliers on the prediction accuracies by performing two additional analyses and checking the consistency of the results. In the first additional analysis, the CES-D scores were transformed using a Box–Cox transformation. In the second additional analysis, we excluded the CES-D scores that were outliers. The outliers were detected based on boxplots adjusted for skewed distributions^28^. The samples with CES-D scores of 0 or greater than 33 (3.5% and 4.7% in the training and test datasets, respectively) were determined to be outliers and excluded in this additional analysis.

The demographics of the members of each dataset are shown in Table 1. Because the training and validation cohorts were recruited independently, the percentage of females, age, educational background, house damage from the 2011 Great East Japan Earthquake and Tsunami, and the time between the disaster and the measurement of CES-D significantly differed between cohorts, which could have made genetic prediction of depressive state more challenging.

**Table 1:** Demographics of the members of the discovery and validation datasets.

	Discovery dataset	Validation dataset	*P* value^a^
Subjects	3685	3048	
Percent of females	70.1%	65.3%	3.31 × 10^−5^
CES-D, mean (SD)	13.6 (7.2)	13.4 (6.9)	0.226
Age, mean (SD)	58.5 (12.1)	62.0 (10.1)	1.35 × 10^−38^
Educational background			6.54 × 10^−37^
Elementary/junior high school	640 (17.4%)	946 (31.0%)	
High school	1852 (50.3%)	1260 (41.3%)	
Junior college	903 (24.5%)	649 (21.3%)	
College	279 (7.6%)	187 (6.1%)	
Graduate school	11 (0.3%)	6 (0.2%)	
House damage from the 2011 Great East Japan Earthquake and Tsunami			1.09 × 10^−278^
Total collapse	561 (15.2%)	218 (7.2%)	
Large-scale damage	248 (6.7%)	61 (2.0%)	
Half-scale damage	302 (8.2%)	75 (2.5%)	
Small-scale damage	1534 (41.6%)	522 (17.1%)	
No damage	1040 (28.2%)	2172 (71.3%)	
Previous psychiatric history			
Depression	104 (2.8%)	81 (2.7%)	0.708
Bipolar disorder	9 (0.2%)	6 (0.2%)	0.798
Family history^b^			
Depression	203 (5.5%)	167 (5.5%)	1.00
Bipolar disorder	27 (0.7%)	26 (0.9%)	0.583
The gap time between the 2011 Great East Japan Earthquake and measurement of CES-D (months), mean (SD)	28.5 (2.0)	30.8 (1.3)	9.88 × 10^−324^
Prefectures	Miyagi, Japan	Iwate, Japan	

*CES-D* Center for Epidemiologic Studies-Depression Scale, *SD* standard deviation, *GEJE* Great East Japan Earthquake.

^a^*P* values were calculated using Student’s *t* tests for CES-D, age, and the time gap between the 2011 Great East Japan Earthquake and measurement of CES-D and Fisher’s exact tests for the percentage of females, educational background, house damage from the 2011 Great East Japan Earthquake and Tsunami, previous psychiatric history, and family history.

^b^Family history refers to the previous psychiatric history of first-degree relatives (i.e., parents, siblings, or children).

### Outcome measures (simulated phenotypes)

We prepared simulated phenotypes based on our actual SNP data following the previous study^22^, and compared the prediction accuracy of STMGP and those of other prediction models. We set the number of true variants at 100, 200, 500, 2000, and 5000. We set the effect-size distribution for susceptibility SNPs by the normal and Laplace distribution^29,30^. We also considered the normal–exponential gamma (NEG) distribution based on previous studies^31–33^. The NEG distribution can be modeled as a Laplace distribution with a gamma-distributed rate, and it has thicker tails than both the normal and Laplace distributions. Decreasing the gamma-shape parameter leads to thicker tails, whereas increasing this parameter restores the distribution to the Laplace distribution. We set the shape parameter at 2, 3, and 10. In each analysis, 20 replications were performed, and the mean predictive correlation coefficients (PCCs), standard deviations, and *P* values for the PCCs were calculated.

To prepare the phenotype, we randomly selected the above-mentioned number of SNPs that were in approximate linkage equilibrium (*r*^2^ < 0.05). Then, we simulated a phenotype across all individuals, including the training and test datasets, with the selected SNPs as follows:

$y_j = \mathop {\sum}\nolimits_{i = 1}^k {w_{ij}b_i + e_j}$, where $w_{ij} = \frac{{\left( {x_{ij} - 2p_i} \right)}}{{\sqrt {2p_i\left( {1 - p_i} \right)} }}$, with *b*_*i*_ the allelic effect of the *i*th causal variant and *e*_*j*_ the residual (environmental effect) of the *j*th sample. Furthermore, *b*_*i*_ was simulated from the Laplace, normal, or NEG distribution with mean = 0 and variance = 1, and *e*_*j*_ was simulated from a normal distribution with mean = 0 and variance = $\left[ {\sigma _{\mathop {\sum}\nolimits_{i = 1}^k {w_{ij}b_i} }^2 \times \left( {1/h^2 - 1} \right)} \right]$, where *h*^2^ is the heritability of the trait. *h*^2^ was set at 0.05 and 0.10, based on the SNP heritability of CES-D in the current analysis (0.05) and the SNP heritability in the previous large GWAS for depressive phenotype (0.047–0.102)^1–3,20^. Similar to the analysis regarding the CES-D scores, the prediction models were built based on the 3685 training samples and evaluated on 3048 independent validation samples.

### Performance metrics

We estimated the partial correlation coefficients controlling for covariates (age, sex, and principal components) in the models for the prediction of depressive symptoms. The PCC was used for the prediction of simulated phenotypes. To test the significance of the difference between the partial correlation of STMGP and those of the tested methods, we used William’s test^34^, which tests the difference between two dependent correlations sharing one variable, implemented in the psych package of R.

### Packages and parameters used for prediction models

The program code for STMGP (STMGP v1.0), including the stmgplink function, was available via CRAN, the official R package archive^35^. For the inputs of stmgplink function, we prepared SNP data, phenotype data, and covariate data for both the training and test datasets, as well as two tuning parameters, *τ* and *γ*. STMGP requires individual-level SNP data, not summary statistics, for calculating the correlation between SNPs. The stmgplink function sequentially (1) computed GWAS *P* values, (2) identified an optimal *P*-value cutoff using Mallows’ Cp criterion, (3) built the prediction model from the STMGP model (i.e., computation of the regression coefficients for SNPs by weighting based on GWAS test statistics and correlations among SNPs in a generalized ridge-regression model with the weights) of the training dataset, and (4) predicted the phenotypes in the test dataset^15^.

In addition to the *P*-value cutoff, there are two tuning parameters for STMGP, *τ* and *γ*. *τ* controls the extent of the overall penalization. Specifically, *τ* regulates the loss function ||*y*−*X*_A_*β*_A_||^2^, where *y*, *X*_A_, and *β*_A_ indicate the vector of the phenotype, the matrix of the predictor variables (standardized allele numbers of screened SNPs), and the vector of the regression coefficients. A denotes the set of selected SNPs (i.e., the nonnull variants) at a given *P*-value cutoff. Thus, *τ* must be adjusted depending on the sample size (*N*) because the loss function is the residual sum of squares, and thus increases proportionally to *N* when *N* is large. The previous study by Ueki and Tamiya^15^ suggested $N/\sqrt {{\mathrm{log}}(N)}$ as a τ parameter based on a study of simulated and real genomic data. In this study, in addition to the main analysis setting *τ* equal to $N/\sqrt {{\mathrm{log}}(N)}$, additional analyses in which *τ* was set to *N*/0.1, *N*/1, and *N*/10 were also performed.

*γ* controls the influence of the input GWAS test statistics on the SNP weight for the generalized ridge-regression model, and the magnitude of the *P* value is incorporated by smooth thresholding rather than the hard thresholding utilized in PRS. Because of the equivalence to the *γ* parameter in the adaptive Lasso^36^ shown in ref. ^16^, we set *γ* to the commonly used value 1 for the adaptive Lasso used herein^37–40^.

Packages and parameters used for genetic predictions other than STMGP are shown in the Supplementary Methods section.

### Covariate adjustment for STMGP

The covariates of sex, age, and the significant principal components to control population stratification were included in the prediction models. The principal component analyses were performed for the SNP data, including the training and test data (6733 subjects), and the *P* values for all the principal components (i.e., the first principal component ~the 6733rd principal component) were calculated based on the Tracy–Widom distribution using the Eigensoft package^41,42^. The components with *P* values < 0.05 (i.e., the first principal component ~the 26th component) were used as covariates. The SNP data, including the training and test datasets, were used for calculating the principal components, to ensure that the same set of principal components was used for the training and test datasets. The scatterplot generated by principal component analysis is shown in Supplementary Fig. 2. The prediction model including covariates was trained from the training samples by the following model:

1 $$y_{{\mathrm{train}}} = b_0 + b_1 \times {\mathrm{AGE}}_{{\mathrm{train}}} + b_2 \times {\mathrm{SEX}}_{{\mathrm{train}}} + b_3 \times PC1_{{\mathrm{train}}} + b_4 \times PC2_{{\mathrm{train}}} + \cdots + {{{\mathbf{SNP}}}}_{{\mathrm{train}}}{\boldsymbol{b}}_{{\mathrm{SNP}}} + e_{{\mathrm{train}}}$$

The regression coefficients (*b*_0_, *b*_1_, *b*_2_, *b*_3_, *b*_4_, … ***b***_**SNP**_) were obtained by the STMGP method, and the values were used to calculate the predicted score of each test sample by the following formula:

2 $$\mu _{{\mathrm{test}}} = b_0 + b_1 \times {\mathrm{AGE}}_{{\mathrm{test}}} + b_2 \times {\mathrm{SEX}}_{{\mathrm{test}}} + b_3 \times PC1_{{\mathrm{test}}} + b_4 \times PC2_{{\mathrm{test}}} + \cdots + {{{\mathbf{SNP}}}}_{{\mathrm{test}}}{\boldsymbol{b}}_{{\mathrm{SNP}}}$$

in which *y*, AGE, and SEX represent an individual’s phenotypic score (CES-D), age, and sex, and PC1, PC2, … are the principal components that must be adjusted for. *e*_train_ is an error term. **SNP** represents the vector of the standardized allele number of the selected SNPs. Covariate adjustment for genetic predictions other than STMGP is shown in the Supplementary Methods section.

Because the method for adjusting for confounding covariates can affect the prediction accuracy, we investigated the consistency among the results under different methods of handling the covariates. We considered three additional approaches in addition to calculating the partial correlation coefficient adjusted for age, sex, and principal components. In the first approach, we regressed out age and sex, and evaluated the prediction accuracy of our genetic scores against the residuals of the regression. In the second approach, we calculated the predictive correlation coefficient without adjusting for the covariates. The third approach was the inclusion of the degree of damage from the 2011 Great East Japan Earthquake and Tsunami into the covariates, because depressive phenotypes may have been affected by this event^43–45^. The damage was coded based on house damage as determined by the local government^43^ following the national damage certification standards of disasters: 4 = total collapse (uninhabitable), 3 = large-scale damage (requires major repairs), 2 = half-scale damage (habitable with repairs), 1 = small-scale damage, and 0 = no damage.

### Evaluation of the SNPs selected by STMGP

To investigate the relationship between the SNPs selected by STMGP and the risk alleles suggested by previous GWAS studies, we referred to the results from the genome-wide meta-analysis by Howard et al.^3^. This genome-wide meta-analysis was one of the largest meta-analyses related to depression, and included 170,756 cases and 329,443 controls, from 33 cohorts of the Psychiatric Genomics Consortium as described in Wray et al.^2^ and the broad depression phenotype in the full release of the UK Biobank as described in Howard et al.^20^.

We first selected the proxy SNPs from phase 3 of the 1000 Genome Project^46,47^ within a 100-kilobase window around the 102 SNPs selected by STMGP using proxysnps software (https://github.com/slowkow/proxysnps). Then, we calculated *r*^2^ values between these proxy SNPs and the SNPs included in STMGP, and we checked the *P* values of the proxy SNPs in the previous genome-wide meta-analysis^3^. To evaluate the MAFs of the SNPs used in the STMGP algorithm among different ethnic groups, the Genome Aggregation Database (gnomAD)^48^ was utilized.

## Results

We first evaluated the performance of the models in predicting depressive symptoms. When the prediction accuracies of all conducted models were calculated using the independent validation cohort, the STMGP prediction model showed the highest partial correlation (Table 2), but it was not significantly different from that of the other prediction models (*P* values > 0.05). When the training cohort was used both for building the model and calculating the prediction accuracy, i.e., the apparent (resubstitution) prediction accuracy, the partial correlation of STMGP was less optimistic than that of the other models, and the degree of overfitting successfully decreased in STMGP (Table 2). The Manhattan plot and QQ plot of GWAS are shown in Supplementary Fig. 3. The computational time and consumed memory at peak time for the STMGP calculations were 107 min and 13 GB, respectively, which can be handled by common computer servers.

**Table 2:** Prediction accuracy for depressive states.

	Partial correlations in the independent validation datasets (SE)	*P* value	Partial correlations in the training datasets (SE)	Number of variants included in prediction models
STMGP	0.0530 (0.0180)	3.424 × 10^−3^	0.3230 (0.0151)	102
PRS	0.0247 (0.0178)	0.1724	0.9025 (0.0076)	13,421
GBLUP	0.0211 (0.0178)	0.2431	0.9623 (0.0017)	601,239
SBLUP	0.0134 (0.0178)	0.3663	0.9554 (0.0019)	599,149
BayesR	0.0190 (0.0185)	0.2871	0.9633 (0.0015)	615,386
Ridge	0.0160 (0.0178)	0.4321	0.9998 (0.0000)	30,333

*PCC* predictive correlation coefficient, *SE* standardized error, *STMGP* Smooth-Threshold Multivariate Genetic Prediction, *PRS* polygenic risk scores, *GBLUP* genomic best linear-unbiased prediction, *SBLUP* summary-data-based best linear-unbiased prediction, *SNP* single-nucleotide polymorphism, *PC* principal component.

Partial correlations were adjusted by covariates such as sex, age, and PC1 ~26.

Since ridge regression based on raw SNP data was difficult to implement in our environment due to the substantial computational cost, the genome data were clumped into approximately 30,000 SNPs in a manner similar to a previous study for these analyses^51^.

Information about the variants used for STMGP and the related information from a previously published genome-wide meta-analysis^3^ are shown in Supplementary Table 1 and Supplementary Fig. 4. Among the *P* values from the previous meta-analysis on the SNPs in strong LD (*r*^2^ > 0.8) with the SNPs selected by STMGP, the smallest *P* value was 0.00112 (rs2678198). The highest *r*^2^ value between the SNPs selected by STMGP and the risk alleles suggested by the previous meta-analysis (*P* value < 5e–5) was 0.0722 (rs4977974 and rs1758737). Information about ethnicity for the selected 102 SNPs is shown in Supplementary Fig. 5 and Supplementary Table 2.

The *P*-value cutoff obtained by STMGP was 2.7 × 10^−4^, which was lower than the *P*-value cutoff obtained by PRS (0.022). We also analyzed PRS using the same cutoff of that used for STMGP, and the partial correlation of this modified PRS was 0.0230, which was not higher than that of the original PRS (0.0247).

The slope of the regression of the phenotype (CES-D) on predicted values was calculated to compare the difference in the predicted score and the difference in the phenotype. The slope values of the regressions (SE) for STMGP, PRS, GBLUP, SBLUP, BayesR, and ridge regression were 0.591 (0.137), 0.100 (0.055), 0.097 (0.055), 0.063 (0.057), 0.050 (0.055), and 0.050 (0.033), respectively. The slope of the regression and scatterplots of the true and predicted scores are shown in Supplementary Fig. 6. The regression of STMGP was closer to 1 than other prediction models, which showed that STMGP would be a less-biased predictor and useful when combined with different information for prediction^23^.

The prediction accuracies of STMGP using different *τ* parameters are shown in Supplementary Table 3. The prediction power was still higher than that of the competitors over various *τ* values, and setting *τ* = *N*/10 showed the best partial correlation (0.0964) in the current datasets. The performance of the models predicting the phenotype with different distribution (Box–Cox-transformed phenotype/the outlier-excluded dataset) is shown in Supplementary Table 4. The prediction accuracies of alternative methods of handling covariates (regressing out the covariates, using damage from the Great East Japan Earthquake, and PCC not adjusted for covariates) are shown in Supplementary Tables 5 and 6. The performance of the prediction models based on the imputed genome datasets is shown in Supplementary Table 7. Although the partial correlation of STMGP based on the imputed genome data (0.0628) was not significantly higher than the partial correlation of STMGP based on raw SNP data (0.0530, *P* value > 0.05), STMGP showed better prediction accuracy than the other methods by decreasing overfitting.

Following the prediction analyses for depressive symptoms, studies using simulated phenotypes based on the current SNP data were performed to evaluate the performance of STMGP in predicting phenotypes with varying complexities and various effect-size distributions of risk SNPs. The results of the simulation studies (the risk SNP effect-size distribution was based on the normal and Laplace distributions) are shown in Tables 3 (heritability = 0.05) and 4 (heritability = 0.10). The results from the scenarios that set the effect-size distribution based on the NEG distribution are shown in Supplementary Tables 8 and 9. The STMGP tended to have better prediction accuracy than the other prediction models in scenarios in which (i) the SNP effect-size distribution was based on the Laplace or NEG distribution, not the normal distribution, and (ii) the complexity of the phenotype was moderate (2000 or fewer-risk SNPs).

**Table 3:** Prediction accuracy in simulation studies in which the phenotype is associated with SNPs only (heritability = 0.05).

Distribution of the true SNP effects	Prediction models	Number of true susceptibility variants									
		100		200		500		2000		5000	
		Mean (SE) PCC	Power^a^	Mean (SE) PCC	Power^a^	Mean (SE) PCC	Power^a^	Mean (SE) PCC	Power^a^	Mean (SE) PCC	Power^a^
Laplace distribution	STMGP	0.0594 (0.0243)	0.85	0.0440 (0.0311)	0.65	−0.0044 (0.0215)	0.15	0.0143 (0.1939)	0.15	−0.0028 (0.0135)	0.00
	PRS	0.0089 (0.0240)	0.15	0.0094 (0.0187)	0.10	−0.0070 (0.0208)	0.15	0.0059 (0.0173)	0.05	−0.0017 (0.0133)	0.05
	GBLUP	0.0118 (0.0159)	0.05	0.0080 (0.0155)	0.00	0.0067 (0.0210)	0.15	0.0149 (0.0013)	0.05	0.0160 (0.0142)	0.10
	SBLUP	0.0048 (0.0140)	0.00	0.0100 (0.0137)	0.00	0.0083 (0.0198)	0.10	0.0142 (0.0129)	0.05	0.0111 (0.0193)	0.10
	BayesR	0.0391 (0.0494)	0.65	0.0264 (0.0273)	0.30	0.0073 (0.0234)	0.15	0.0144 (0.0142)	0.10	0.0109 (0.0176)	0.10
	Ridge	0.0052 (0.0146)	0.05	0.0049 (0.0155)	0.00	0.0104 (0.0216)	0.15	0.0085 (0.0132)	0.05	0.0072 (0.0172)	0.00
Normal distribution	STMGP	0.0475 (0.0238)	0.70	0.0140 (0.0170)	0.10	0.0082 (0.0197)	0.15	0.0112 (0.0071)	0.10	0.0040 (0.0175)	0.05
	PRS	0.0028 (0.0207)	0.05	0.0054 (0.0191)	0.15	0.0017 (0.0185)	0.05	−0.0011 (0.0189)	0.05	0.0031 (0.0146)	0.10
	GBLUP	0.0120 (0.0135)	0.05	0.0103 (0.0171)	0.05	0.0133 (0.0147)	0.10	0.0127 (0.0199)	0.10	0.0130 (0.0154)	0.10
	SBLUP	0.0117 (0.0177)	0.15	0.0109 (0.0167)	0.05	0.0057 (0.0145)	0.10	0.0068 (0.0155)	0.00	0.0116 (0.0127)	0.05
	BayesR	0.0239 (0.0271)	0.35	0.0147 (0.0185)	0.10	0.0073 (0.0168)	0.10	0.0108 (0.0194)	0.05	0.0092 (0.0133)	0.05
	Ridge	0.0144 (0.0162)	0.20	0.0135 (0.0170)	0.05	0.0083 (0.0185)	0.00	0.0100 (0.0197)	0.05	0.0075 (0.0187)	0.00

*PCC* predictive correlation coefficient, *SE* standardized error, *STMGP* Smooth-Threshold Multivariate Genetic Prediction, *PRS* polygenic risk scores, *GBLUP* genomic best linear-unbiased prediction, *SBLUP* summary-data-based best linear-unbiased prediction, *NEG* normal–exponential–gamma.

^a^Power is the proportion of replicates achieving a significant prediction at *P* value < 0.05.

## Discussion

The current study showed that STMGP is useful for predicting psychiatric polygenic phenotypes. In predicting depressive phenotypes, STMGP showed the highest prediction accuracy, and in the simulation study, STMGP tended to have better performance in predicting moderately polygenic phenotypes. The strategy of STMGP (i.e., screening and building penalized regression models) successfully reduced overfitting. The computational cost of STMGP was relatively low for our imputed genome data.

**Table 4:** Prediction accuracy in simulation studies in which the phenotype is associated with SNPs only (heritability = 0.10).

Distribution of the true SNP effects	Prediction models	Number of true susceptibility variants									
		100		200		500		2000		5000	
		Mean (SE) PCC	Power^a^	Mean (SE) PCC	Power^a^	Mean (SE) PCC	Power^a^	Mean (SE) PCC	Power^a^	Mean (SE) PCC	Power^a^
Laplace distribution	STMGP	0.1520 (0.0293)	1.00	0.1029 (0.0408)	1.00	0.0521 (0.0252)	0.80	0.0241 (0.0193)	0.35	0.0217 (0.0171)	0.25
	PRS	0.0454 (0.0434)	0.75	0.0421 (0.0247)	0.85	–0.0018 (0.0283)	0.10	0.0128 (0.0203)	0.15	0.0004 (0.0203)	0.10
	GBLUP	0.0137 (0.0134)	0.05	0.0201 (0.0143)	0.15	0.0163 (0.0190)	0.20	0.0198 (0.0133)	0.25	0.0199 (0.0201)	0.15
	SBLUP	0.0140 (0.0148)	0.05	0.0186 (0.0143)	0.10	0.0150 (0.0200)	0.20	0.0189 (0.0159)	0.25	0.0186 (0.0189)	0.15
	BayesR	0.1217 (0.0680)	0.90	0.0782 (0.0475)	0.85	0.0345 (0.0337)	0.35	0.0202 (0.0195)	0.25	0.0172 (0.0222)	0.15
	Ridge	0.0183 (0.0158)	0.20	0.0188 (0.0138)	0.20	0.0215 (0.0212)	0.30	0.0184 (0.0111)	0.10	0.0171 (0.0192)	0.15
Normal distribution	STMGP	0.1045 (0.0281)	1.00	0.0638 (0.0205)	0.95	0.0236 (0.0122)	0.30	0.0208 (0.0156)	0.25	0.0195 (0.0186)	0.15
	PRS	0.0258 (0.0305)	0.50	0.0177 (0.0220)	0.30	0.0079 (0.0224)	0.15	0.0053 (0.0216)	0.10	0.0015 (0.0233)	0.00
	GBLUP	0.0220 (0.0168)	0.30	0.0202 (0.0172)	0.15	0.0161 (0.0147)	0.15	0.0172 (0.0191)	0.15	0.0204 (0.0132)	0.15
	SBLUP	0.0215 (0.0173)	0.30	0.0195 (0.0174)	0.15	0.0173 (0.0150)	0.15	0.0185 (0.0198)	0.20	0.0206 (0.0129)	0.15
	BayesR	0.0943 (0.0489)	0.90	0.0444 (0.0224)	0.70	0.0210 (0.0171)	0.15	0.0189 (0.0135)	0.20	0.0130 (0.0127)	0.05
	Ridge	0.0251 (0.0156)	0.40	0.0269 (0.0180)	0.40	0.0187 (0.0184)	0.15	0.0170 (0.0162)	0.15	0.0154 (0.0179)	0.10

*PCC* predictive correlation coefficient, *SE* standardized error, *STMGP* Smooth-Threshold Multivariate Genetic Prediction, *PRS* polygenic risk scores, *GBLUP* genomic best linear-unbiased prediction, *SBLUP* summary-data-based best linear-unbiased prediction, *NEG* normal–exponential–gamma.

^a^Power is the proportion of replicates achieving a significant prediction at *P* value < 0.05.

The prediction for depressive symptoms in the current datasets was a challenging situation, with low prediction correlations for all state-of-the-art methods, in which only STMGP showed significant prediction accuracy, but the difference in accuracy between STMGP and the other models was not significant. The largest standardized regression coefficient of the susceptibility variants, which was calculated by PLINK using the standard beta option, for the depressive phenotype in the current study, was only 0.057. The GWAS results in the training dataset of the current study yielded no genome-wide significant variants (*P* value < 5 × 10^−8^), with only 11 variants with *P* value < 5 × 10^−5^. The top 11 variants explained only 3.6 × 10^−3^% of the variance in the target phenotype in the validation dataset. This small effect size of the susceptibility variants and difficulty in genetic prediction for depression were consistent with previous findings^1,2,14^, which may confirm that depression is one of the most difficult target diseases for genetic prediction. Even in such a challenging situation, STMGP successfully avoided overfitting and yielded the highest prediction accuracy.

Following the prediction for depressive phenotype, we investigated the predictive performance of STMGP using simulated phenotypes with various complexities and different effect-size distributions of risk SNPs. STMGP tended to have better performance compared with other prediction models when predicting the phenotype with moderate complexity (number of susceptible SNPs ≦ 2000 and Laplace or NEG distribution), which could be due to the following reasons. First, the heritability was set to a low value (0.05 or 0.10) in the simulation study, and the effect size of each risk SNP was so small in the highly complex phenotypes that any prediction model, including STMGP, could not effectively use the genetic effects for prediction. Second, the strategy of STMGP to screen SNPs and to adaptively penalize the regression coefficients of each SNP depending on the effect size would be more effective in predicting phenotypes in which the SNP effects have a heavy-tailed distribution than a normal distribution. Considering the finding that STMGP tends to have better performance in moderately polygenic scenarios, it is possible that STMGP could also have good prediction accuracy in predicting less polygenic psychiatric conditions than depression (e.g., schizophrenia and bipolar disorder)^49^. As a result, we are planning a study using STMGP to predict other psychiatric phenotypes.

When the slopes of the regression of the phenotype for the predicted values were evaluated, the slope of the regression (SE) with STMGP was 0.591 (0.137), and those with the other prediction models were even lower. These relatively low slope values would be due to low prediction accuracy rather than bias of the prediction models because the slope values calculated in the training datasets were close to 1 in all the models (0.959–1.032) (Supplementary Fig. 6). It is also possible that the low slope values were due to outliers in the datasets. The regression slope of GBLUP was 0.097 in the current test datasets, but 0.351 in outlier-excluded test datasets, which is close to the slope value in the previous study to predict major depression disorder based on GBLUP models (0.304)^14^.

This study was intended to compare the STMGP algorithm with other prediction models, rather than to discuss the significance of the associations between depression and the individual SNPs selected by STMGP. Although the selected SNPs may have included SNPs that were associated with the CES-D in both independent cohorts in this study after controlling for population stratification, the *P* value of each SNP in the GWAS in the training datasets was relatively large, and no SNPs were genome-wide significant. Furthermore, the selected SNPs did not show strong linkage disequilibrium with the risk SNPs suggested by the largest meta-analysis in Europe. This could have been due to the differences in phenotype (i.e., depression and CES-D), ethnicity (i.e., European and Japanese), and a limited sample size. In fact, the MAFs of the selected SNP were substantially different between the East Asian and European populations. This study succeeded in showing superior performance of STMGP compared with the other prediction models in the current datasets in which all the participants were Japanese. To apply the STMGP model to a different dataset with different ethnicities, such as European and African samples, updates for the training data would be needed, i.e., including samples of close ethnicity to the target population.

The SNP heritability estimated by GREML based on 3,685 training samples was calculated to be 0.05 (SE 0.07) in this study. This heritability value was consistent with the result of one of the largest genome-wide association meta-analyses using 70,017 subjects and depression scores (SNP heritability of 0.04 (SE = 0.01))^21^. The higher SE in the current study relative to that in the previous meta-analyses could be due to the smaller sample size.

The expected prediction accuracy could in theory (i.e., the proportion of phenotypic variance explained by all SNPs based on linear mixed models) be calculated to be 6.61 × 10^−3^% (PCC = 8.13 × 10^−3^)^50^ if all marker effects are assumed to come from the same normal distribution. The STMGP showed better prediction accuracy than the above-mentioned theoretical prediction accuracy, which suggested that the generalized ridge-regression models would better fit the current genome and phenotype data than linear mixed models.

BayesR was developed with a similar rationale as STMGP, which tries to reduce the inclusion of noise, and refine the true association between the phenotype and SNPs by setting multiple mixture distributions of a point mass at zero and normal distribution with different variances. BayesR showed the second highest prediction accuracy in the simulation study based on SNPs only, while the prediction analyses for CES-D scores or the simulation study based on SNPs and covariates did not show the superior performance of BayesR compared with other prediction models. It is possible that our regression models to make use of covariates to increase the prediction accuracy of BayesR were not as effective as with other prediction models with covariate options in the package. Furthermore, it is possible that the sample size was relatively small for BayesR.

There are several limitations related to the study design and the current STMGP implementation. For study design, this study builds and evaluates prediction models based on limited data. The generalization of the results and scalability of prediction models needs to be discussed in the context of larger samples of GWAS data in the future. The lack of information about interventions regarding depression is another limitation of the current study. Information about medication was available only for the discovery cohort at the time of the current analysis. In the discovery cohort, 56 (1.5%) subjects were taking antidepressant drugs. The equivalent number of subjects in the validation cohort may have been taking antidepressant drugs because the prevalence of previous psychiatric history was similar between the discovery and validation cohorts, as shown in Table 1.

There are two limitations to the current STMGP implementation. The STMGP algorithm is essentially a variant of the generalized linear model with an added weighted L2 penalty, meaning that the scalability is comparable with that of the linear/logistic regression with predictor variables screened by a *P*-value cutoff. However, the current implementation is suitable for using a few thousand individuals for training data with an SNP array, imputed genome data, or whole-genome sequencing data. In addition, STMGP can currently handle only individual-level SNP data, not summary statistics, for calculating the correlation between SNPs. We are planning to improve the scalability and develop options for using summary statistics.

In conclusion, this study showed the potential usefulness of STMGP in predicting polygenic psychiatric phenotypes using real GWAS- and simulated data. The strategy to reduce overfitting through screening and building penalized regression models was suggested to be effective in genetic prediction, especially for moderately polygenic phenotypes. Considering its predictive performance and lower computer costs compared with other penalized regression models, STMGP is recommended for the genetic prediction of psychiatric conditions with a polygenic model.

## Supplementary information

Supplementary Methods

Supplementary Figure 1

Supplementary Figure 2

Supplementary Figure 3

Supplementary Figure 4

Supplementary Figure 5

Supplementary Figure 6

Supplementary Table 1

Supplementary Tables 2–7

Supplementary Table 8

Supplementary Table 9
