# Supplementary material for: Machine learning for effectively avoiding overfitting is a crucial strategy for the genetic prediction of polygenic psychiatric phenotypes
Source: Transl Psychiatry. 2020 Aug 17;10:294. doi: 10.1038/s41398-020-00957-5 (PMC7442807; doi:10.1038/s41398-020-00957-5)
Supplement: Supplementary file 1 — Supplementary Methods [file 41398_2020_957_MOESM1_ESM.docx]

Supplementary Methods

Packages and parameters used for genetic predictions other than STMGP

PRS was calculated using the Polygenic Risk Score software PRSice (v1.25), with a default setting of LD-based clumping using the PLINK software, with the option "--clump-p1 1.0 --clump-p2 1.0 --clump-kb 250 --clump-r2 0.1”. A ten-fold cross-validation was conducted with the training data to select the best P-value threshold from successive P-value sets between 5×10^-4^ and 0.5 in increments of 5×10^-4 1^. Genome-wide complex trait analysis (GCTA) v1.26.0 was utilized to perform GBLUP and SBLUP. GBLUP estimates genetic relation matrix using SNP data, estimates the variance explained by all the analyzed variants using restricted maximum likelihood analysis, and predicts random effects from SNPs by the BLUP method. SBLUP is BLUP analysis using summary statistics from GWAS. In SBLUP analysis, cojo-sblup was set to 1.14e7 in the analysis using the raw SNP data and 9.50e7 in the analysis using the imputed genome data. The cojo-wind and thread-num options were set to 1000 and 20, respectively. A Bayesian mixture model was performed with BayesRv2 software, which uses Markov chain Monte Carlo (MCMC) while simultaneously identifying associated SNPs, estimating the genetic variance explained by the SNPs, describing the genetic architecture of the trait and predicting the phenotype from SNP genotypes ^2^. We set the length of the MCMC chain to 50,000 and the number of burnin steps to 20,000, and the SNP effects were updated across 6 SNPs. Then, we set the option of reduced update of “-msize 500 –mrep 5000” to reduce the computational burden, and all SNP effects were only updated for the first 5,000 cycles; thereafter, updating effect size continued until 500 SNPs with nonzero effects had been sampled within a cycle. Ridge regression (penalized regression model) was performed using the glmnet ver2.0-13 package in R. The tuning parameter, λ, in the ridge regression was determined by 10-fold cross validation in the training dataset.

Covariate adjustment for the genetic predictions other than STMGP

The covariate option in the original PRS and GBLUP packages utilizes information regarding age, sex, and the principle components with the goal of merely adjusting for the confounding factors and not of improving prediction accuracy. The packages for SBLUP and BayesR do not have the option to use covariates. Therefore, for PRS, GBLUP, SBLUP, and BayesR, the fixed effects of the covariates were included in the prediction model. For ridge regression, the covariates and SNP were used for prediction, and the prediction model, including the covariates, can be written out using the same equation as STMGP ((1) and (2)).

Preparation of the SNP data for the genetic predictions other than STMGP

For GBLUP and BayesR, the input genome data for each package were SNP data as well as the results of STMGP. Since ridge regression based on raw SNP data or imputed genome data, and SBLUP and BayesR based on imputed genome data were difficult to implement in our environment due to substantial computational cost, the genome data were clumped into approximately 30,000 SNPs in a manner similar to a previous study for these analyses ^3^. Because PRS and SBLUP require summary statistics from GWAS, we performed GWAS using the same covariates as those used in STMGP, and the summary statistics from the GWAS were used for PRS and SBLUP.

Preparation of imputed genotype data and quality control

We used the same genotype data as the main analysis (9,966 Japanese individuals; 4,974 training subjects and 4,992 validation subjects), and these individuals were genotyped by HumanOmniExpressExome BeadChip Array (Illumina Inc., San Diego, CA, USA). Subjects with a low call rate (<0.98) were excluded. After the exclusion of duplicated variants, completely missing variants, and variants with low Hardy-Weinberg equilibrium exact test P-values (<0.05) or low minor allele frequencies (<0.05), the genotype data of 9,961 individuals with the remaining 490,981 variants were phased by SHAPEIT2 (v2. r837) ^4^ with --duohmm -W 5 --thread 16 options. Genotype imputation was performed on the phased genotypes with IMPUTE2 (version 2.3.2) ^5^ with two phased reference panels of (1) 2,049 Japanese individuals from a prospective, general population cohort study performed by the TMM ^6^ and (2) 1000 Genomes Phase 3 data set for East Asians ^7^. The 2,049-member Japanese panel contains 32,507,700 autosomal single-nucleotide variants and short insertion–deletion variants that were obtained as follows: sequencing data sets from HiSeq 2500 were aligned with Bowtie2, version 2.1.0 ^8^; variant calling was performed on the aligned data with Bcftools, version 0.1.17-dev ^9^; and called variants with a P-value from the Hardy-Weinberg equilibrium test of less than 1.00 × 10^–4^ were excluded. For IMPUTE2, we used the following options: -use_prephased_g, -Ne 20000, -align_by_maf_g, and -k_hap 4000. The imputed genotype data in Oxford GEN format were converted to PLINK BED format by selecting the genotype with the highest posterior probability for each SNP and individual. During the conversion, the cases below 0.9 with the highest posterior probability were handled as missing genotypes. We detected 2,156 close relationship pairs (620 in the training cohort and 1,536 in the validation cohort) using the identity-by-descent method implemented in PLINK software (PI_HAT > 0.09375) ^10^ among the discovery cohort, the validation cohort, or between these cohorts. Then, in each of these pairs, the subject with lower call rate was excluded. After the exclusion of the variants with low call rates (<0.99), low Hardy-Weinberg equilibrium exact test P-values (<1x10^-4^) or low minor allele frequencies (<0.01) from the imputed genotype data for 11,030,858 variants, 5,949,462 variants (6,733 subjects including 3,685 training and 3,048 test samples) remained for the downstream analysis.

Reference

1. Euesden J, Lewis CM, O'Reilly PF. PRSice: Polygenic Risk Score software. *Bioinformatics (Oxford, England)* 2015; **31**(9)**:** 1466-1468.

2. Moser G*, et al*. Simultaneous discovery, estimation and prediction analysis of complex traits using a Bayesian mixture model. *PLoS genetics* 2015; **11**(4)**:** e1004969.

3. Cherlin S*, et al*. Prediction of treatment response in rheumatoid arthritis patients using genome-wide SNP data. *Genet Epidemiol* 2018; **42**(8)**:** 754-771.

4. Delaneau O, Howie B, Cox AJ, Zagury JF, Marchini J. Haplotype estimation using sequencing reads. *American journal of human genetics* 2013; **93**(4)**:** 687-696.

5. Howie BN, Donnelly P, Marchini J. A flexible and accurate genotype imputation method for the next generation of genome-wide association studies. *PLoS genetics* 2009; **5**(6)**:** e1000529.

6. Nagasaki M*, et al*. Rare variant discovery by deep whole-genome sequencing of 1,070 Japanese individuals. *Nature communications* 2015; **6:** 8018.

7. Auton A*, et al*. A global reference for human genetic variation. *Nature* 2015; **526**(7571)**:** 68-74.

8. Langmead B, Salzberg SL. Fast gapped-read alignment with Bowtie 2. *Nature methods* 2012; **9**(4)**:** 357-359.

9. Li H, Ruan J, Durbin R. Mapping short DNA sequencing reads and calling variants using mapping quality scores. *Genome research* 2008; **18**(11)**:** 1851-1858.

10. Purcell S*, et al*. PLINK: a tool set for whole-genome association and population-based linkage analyses. *American journal of human genetics* 2007; **81**(3)**:** 559-575.
