# Supplementary material for: Machine learning for effectively avoiding overfitting is a crucial strategy for the genetic prediction of polygenic psychiatric phenotypes
Source: Transl Psychiatry. 2020 Aug 17;10:294. doi: 10.1038/s41398-020-00957-5 (PMC7442807; doi:10.1038/s41398-020-00957-5)
Supplement: Supplementary file 2 — Supplementary Figure 1 [file 41398_2020_957_MOESM2_ESM.pptx]

## Slide 1
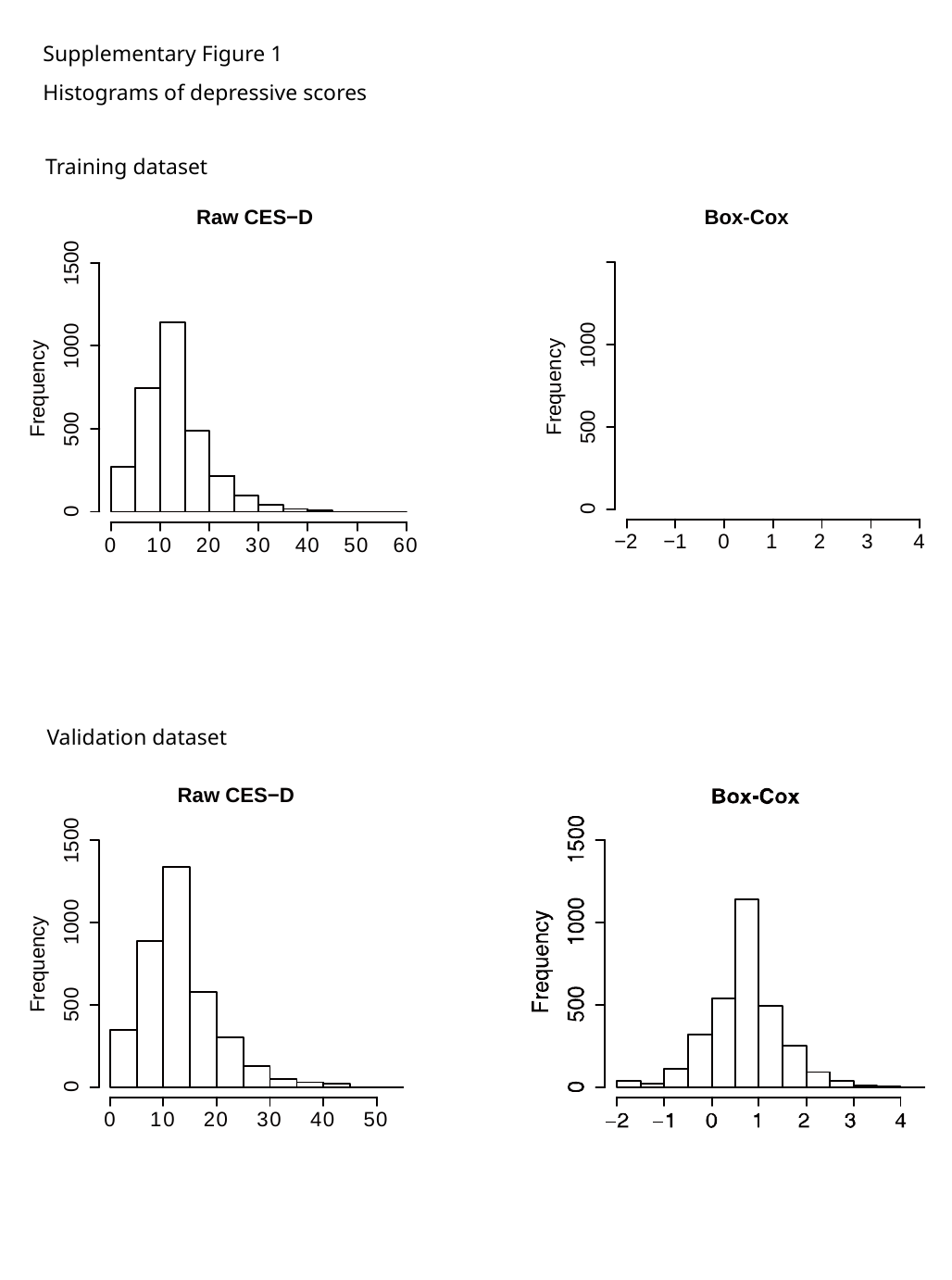

Supplementary Figure 1
Histograms of depressive scores
Training dataset
Validation dataset
