# Supplementary material for: Machine learning for effectively avoiding overfitting is a crucial strategy for the genetic prediction of polygenic psychiatric phenotypes
Source: Transl Psychiatry. 2020 Aug 17;10:294. doi: 10.1038/s41398-020-00957-5 (PMC7442807; doi:10.1038/s41398-020-00957-5)
Supplement: Supplementary file 3 — Supplementary Figure 2 [file 41398_2020_957_MOESM3_ESM.pptx]

## Slide 1
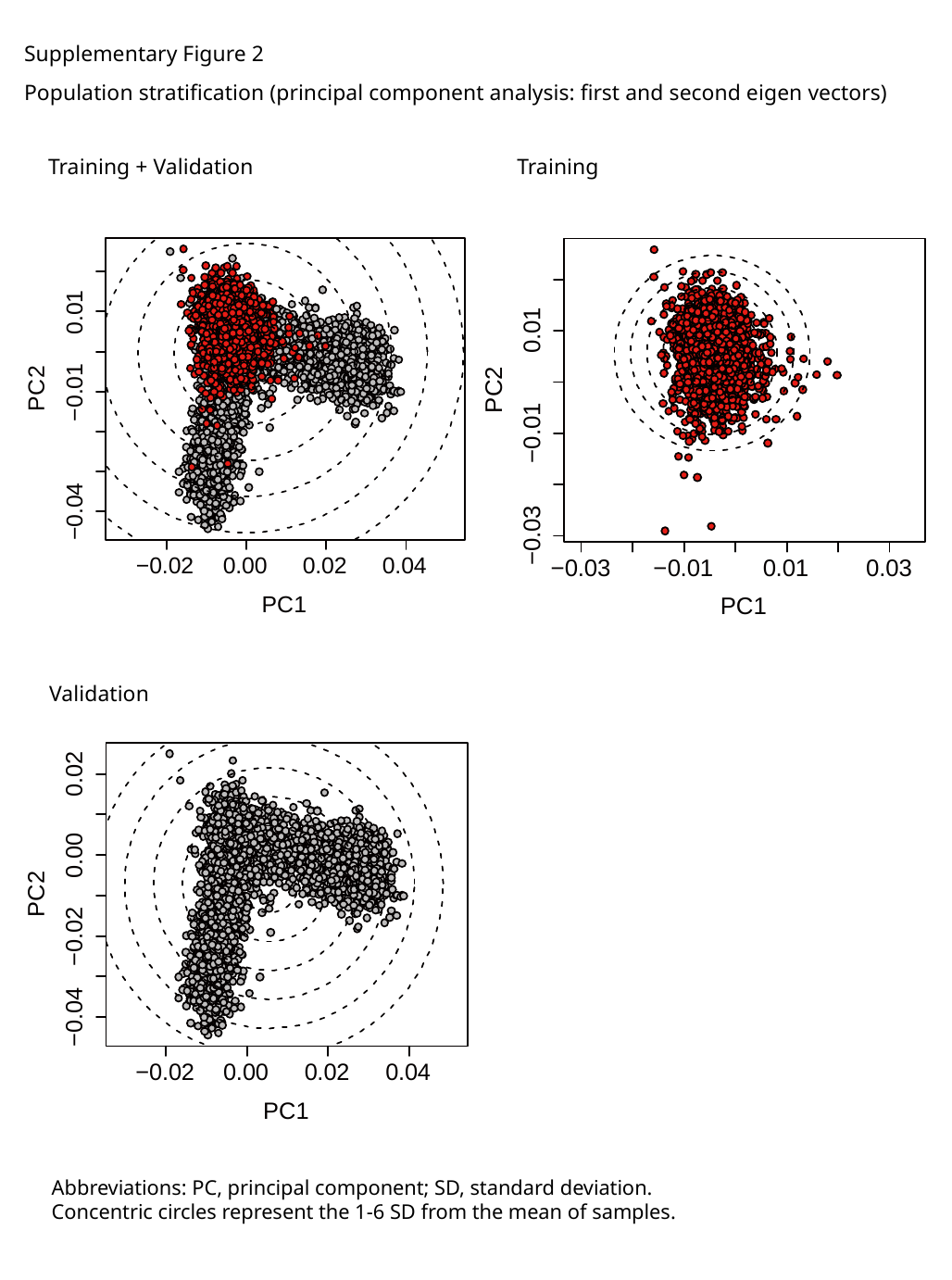

Supplementary Figure 2
Population stratification (principal component analysis: first and second eigen vectors)
Training + Validation
Training
Validation
Abbreviations: PC, principal component; SD, standard deviation.
Concentric circles represent the 1-6 SD from the mean of samples.
