# Supplementary material for: Machine learning for effectively avoiding overfitting is a crucial strategy for the genetic prediction of polygenic psychiatric phenotypes
Source: Transl Psychiatry. 2020 Aug 17;10:294. doi: 10.1038/s41398-020-00957-5 (PMC7442807; doi:10.1038/s41398-020-00957-5)
Supplement: Supplementary file 4 — Supplementary Figure 3 [file 41398_2020_957_MOESM4_ESM.pptx]

## Slide 1
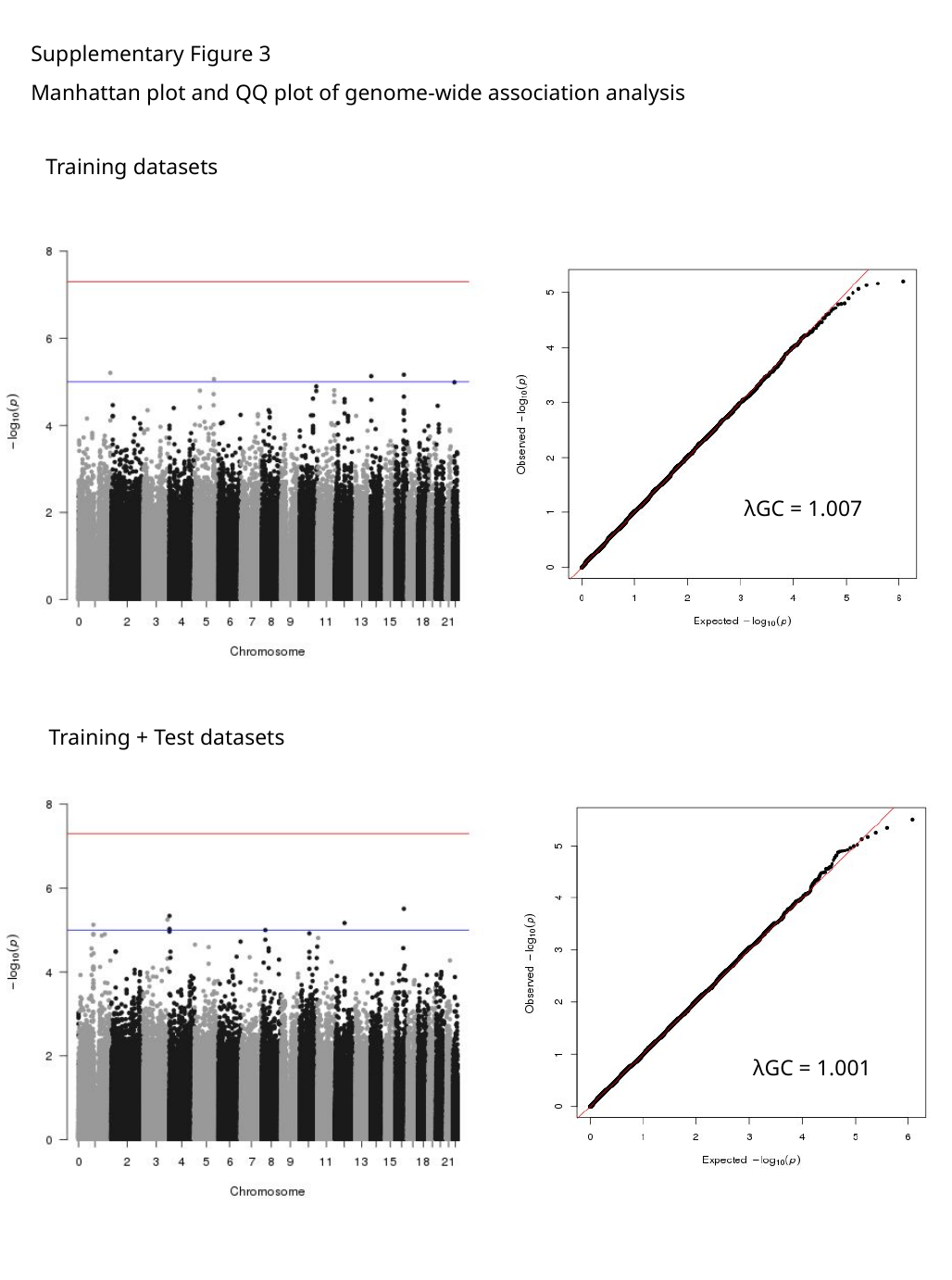

Supplementary Figure 3
Manhattan plot and QQ plot of genome-wide association analysis
Training datasets
λGC = 1.007
Training + Test datasets
λGC = 1.001
