# Supplementary material for: Machine learning for effectively avoiding overfitting is a crucial strategy for the genetic prediction of polygenic psychiatric phenotypes
Source: Transl Psychiatry. 2020 Aug 17;10:294. doi: 10.1038/s41398-020-00957-5 (PMC7442807; doi:10.1038/s41398-020-00957-5)
Supplement: Supplementary file 5 — Supplementary Figure 4 [file 41398_2020_957_MOESM5_ESM.pptx]

## Slide 1
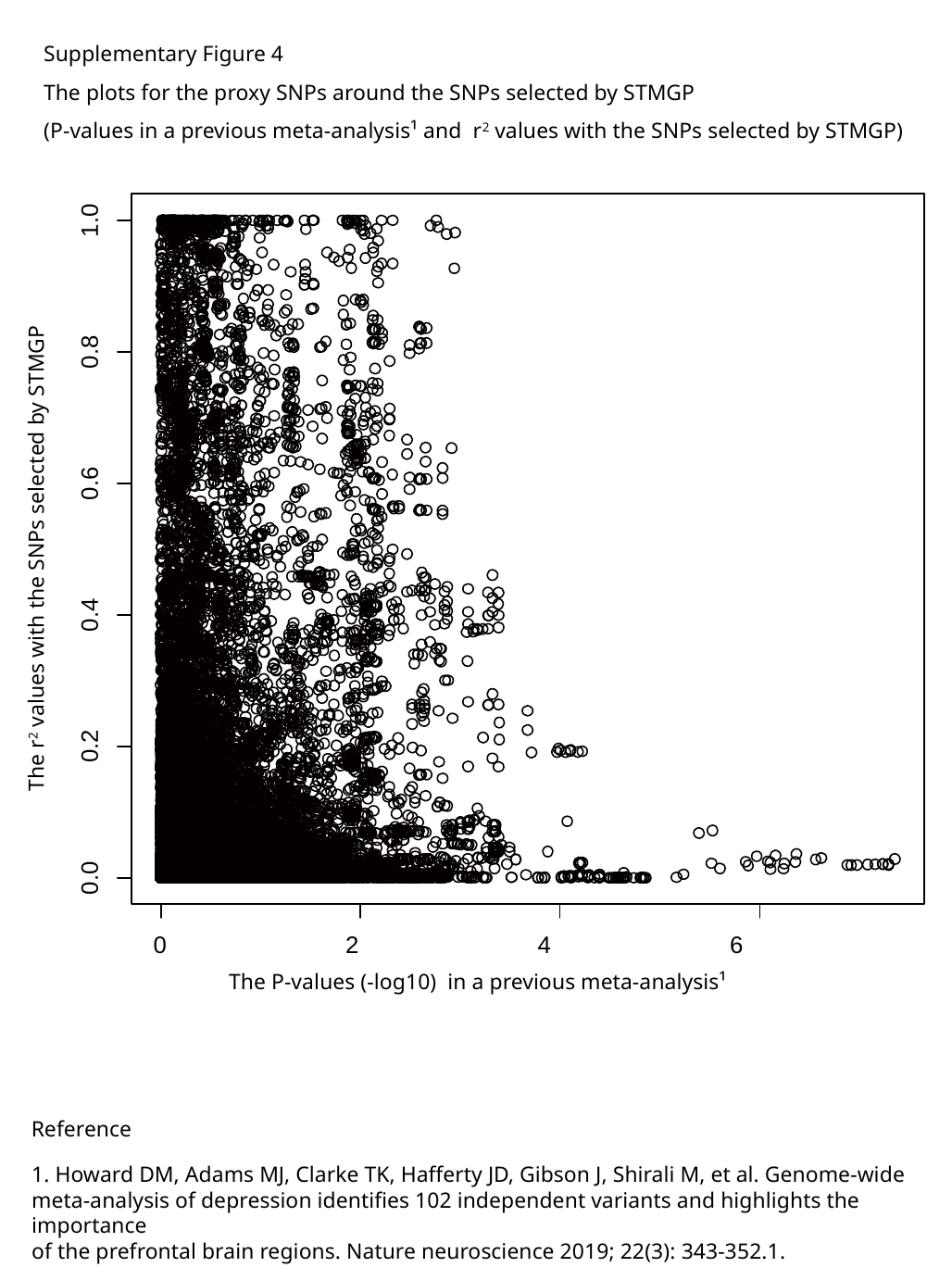

Supplementary Figure 4
The plots for the proxy SNPs around the SNPs selected by STMGP
(P-values in a previous meta-analysis¹ and r2 values with the SNPs selected by STMGP)
The r2 values with the SNPs selected by STMGP
The P-values (-log10) in a previous meta-analysis¹
Reference
1. Howard DM, Adams MJ, Clarke TK, Hafferty JD, Gibson J, Shirali M, et al. Genome-wide
meta-analysis of depression identifies 102 independent variants and highlights the importance
of the prefrontal brain regions. Nature neuroscience 2019; 22(3): 343-352.1.
