# Supplementary material for: Machine learning for effectively avoiding overfitting is a crucial strategy for the genetic prediction of polygenic psychiatric phenotypes
Source: Transl Psychiatry. 2020 Aug 17;10:294. doi: 10.1038/s41398-020-00957-5 (PMC7442807; doi:10.1038/s41398-020-00957-5)
Supplement: Supplementary file 6 — Supplementary Figure 5 [file 41398_2020_957_MOESM6_ESM.pptx]

## Slide 1
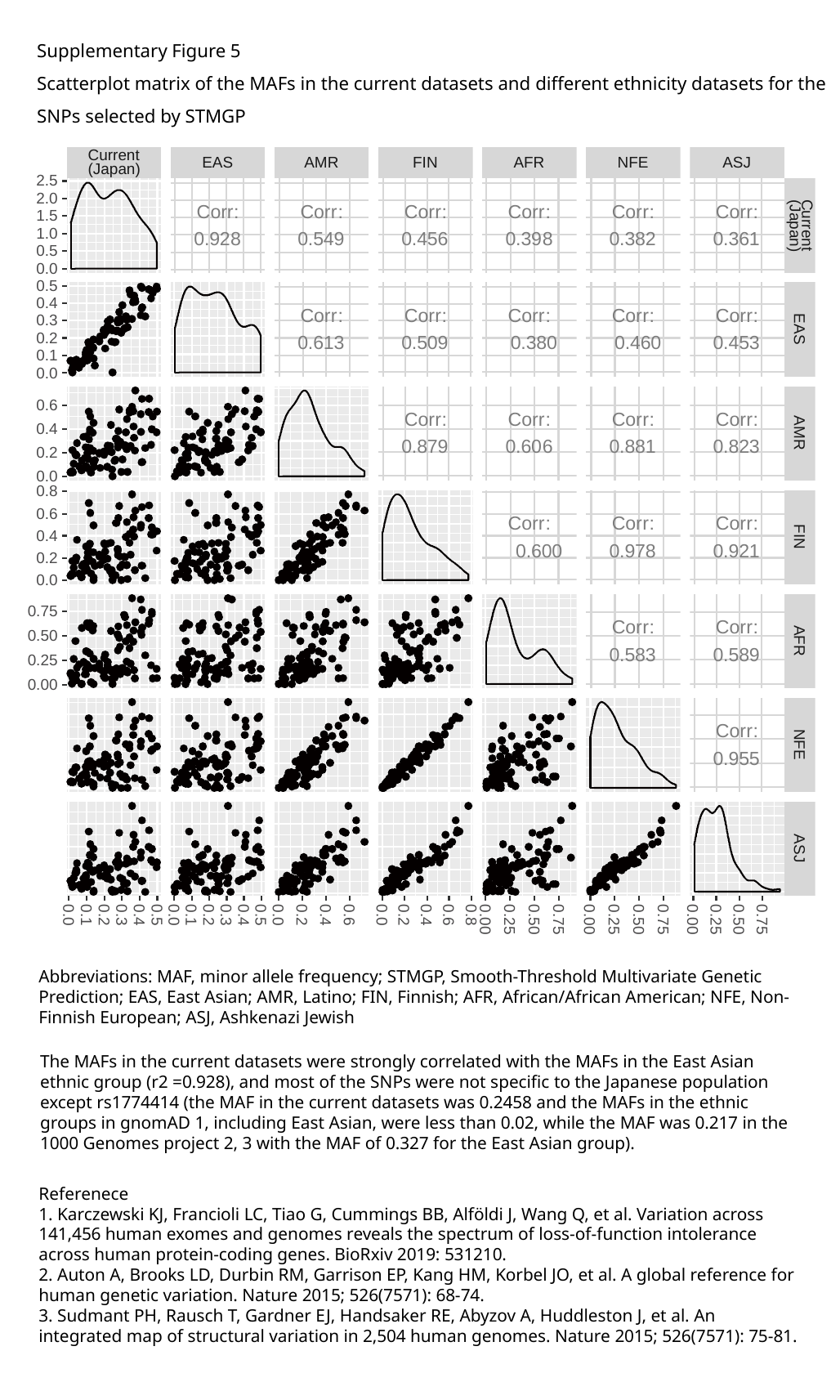

Supplementary Figure 5
Scatterplot matrix of the MAFs in the current datasets and different ethnicity datasets for the
SNPs selected by STMGP
Abbreviations: MAF, minor allele frequency; STMGP, Smooth-Threshold Multivariate Genetic Prediction; EAS, East Asian; AMR, Latino; FIN, Finnish; AFR, African/African American; NFE, Non-Finnish European; ASJ, Ashkenazi Jewish
The MAFs in the current datasets were strongly correlated with the MAFs in the East Asian ethnic group (r2 =0.928), and most of the SNPs were not specific to the Japanese population except rs1774414 (the MAF in the current datasets was 0.2458 and the MAFs in the ethnic groups in gnomAD 1, including East Asian, were less than 0.02, while the MAF was 0.217 in the 1000 Genomes project 2, 3 with the MAF of 0.327 for the East Asian group).
Referenece
1. Karczewski KJ, Francioli LC, Tiao G, Cummings BB, Alföldi J, Wang Q, et al. Variation across 141,456 human exomes and genomes reveals the spectrum of loss-of-function intolerance across human protein-coding genes. BioRxiv 2019: 531210.
2. Auton A, Brooks LD, Durbin RM, Garrison EP, Kang HM, Korbel JO, et al. A global reference for human genetic variation. Nature 2015; 526(7571): 68-74.
3. Sudmant PH, Rausch T, Gardner EJ, Handsaker RE, Abyzov A, Huddleston J, et al. An integrated map of structural variation in 2,504 human genomes. Nature 2015; 526(7571): 75-81.
