# Supplementary material for: Machine learning for effectively avoiding overfitting is a crucial strategy for the genetic prediction of polygenic psychiatric phenotypes
Source: Transl Psychiatry. 2020 Aug 17;10:294. doi: 10.1038/s41398-020-00957-5 (PMC7442807; doi:10.1038/s41398-020-00957-5)
Supplement: Supplementary file 7 — Supplementary Figure 6 [file 41398_2020_957_MOESM7_ESM.pptx]

## Slide 1
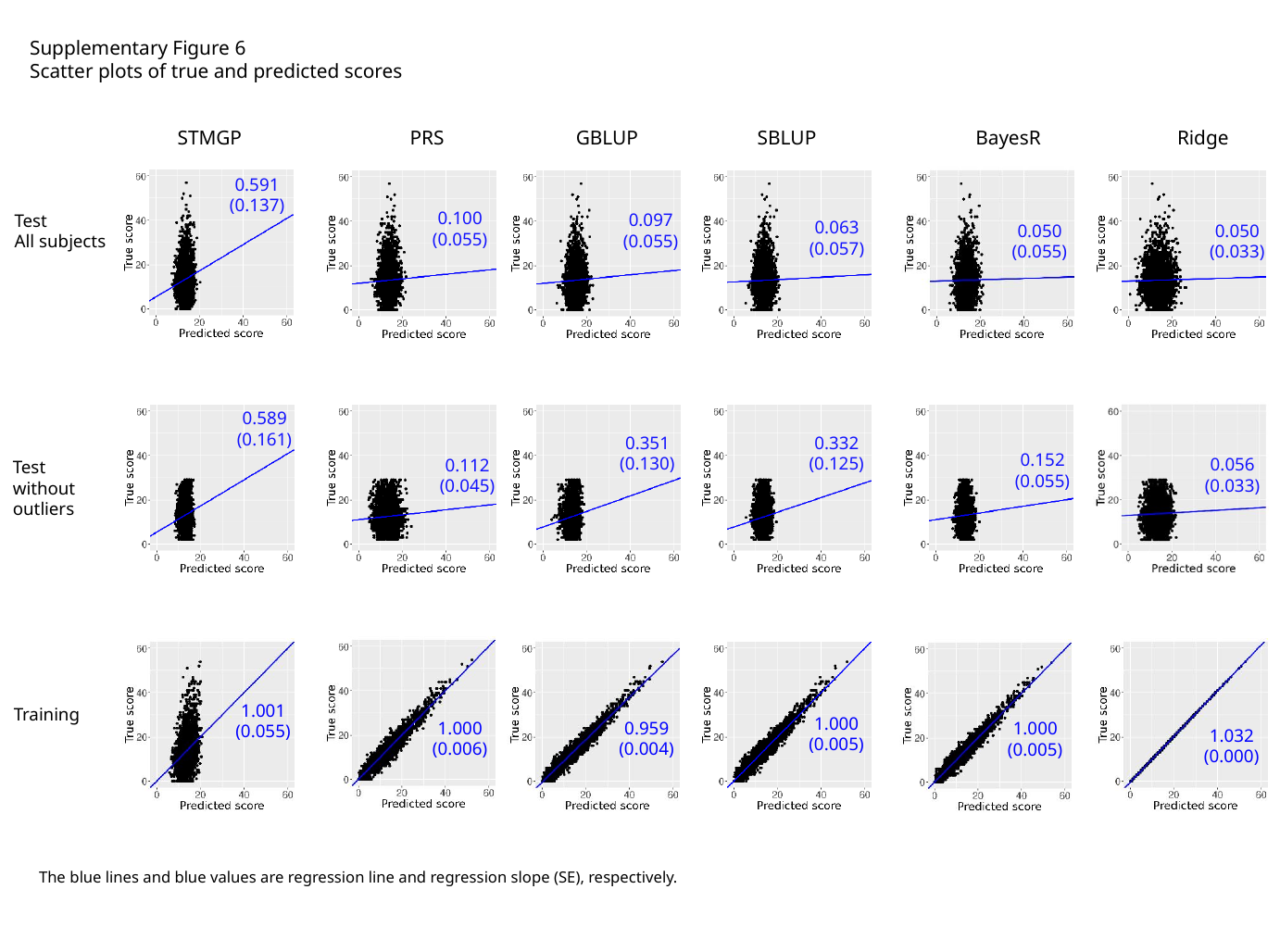

Supplementary Figure 6
Scatter plots of true and predicted scores
STMGP
PRS
GBLUP
SBLUP
BayesR
Ridge
0.591
(0.137)
0.100
(0.055)
0.097
(0.055)
Test
All subjects
0.063
(0.057)
0.050
(0.055)
0.050
(0.033)
0.589
(0.161)
0.351
(0.130)
0.332
(0.125)
0.152
(0.055)
0.056
(0.033)
0.112
(0.045)
Test
without
outliers
1.001
(0.055)
Training
1.000
(0.005)
1.000
(0.006)
0.959
(0.004)
1.000
(0.005)
1.032
(0.000)
The blue lines and blue values are regression line and regression slope (SE), respectively.
