# Supplementary material for: Machine learning for effectively avoiding overfitting is a crucial strategy for the genetic prediction of polygenic psychiatric phenotypes
Source: Transl Psychiatry. 2020 Aug 17;10:294. doi: 10.1038/s41398-020-00957-5 (PMC7442807; doi:10.1038/s41398-020-00957-5)
Supplement: Supplementary file 9 — Supplementary Tables 2–7 [file 41398_2020_957_MOESM9_ESM.docx]

Supplementary Table 2

The proportion of rare variants (MAF<0.05) in each dataset and the proportion shared in the two ethnic groups among the 102 SNPs selected in STMGP

|  | Current (Japanese) | EAS | AMR | FIN | AFR | NFE | ASJ |
| --- | --- | --- | --- | --- | --- | --- | --- |
| Current (Japanese) | 0.12 | 0.05 | 0.06 | 0.03 | 0.03 | 0.00 | 0.00 |
| EAS | - | 0.08 | 0.04 | 0.01 | 0.03 | 0.01 | 0.01 |
| AMR | - | - | 0.10 | 0.06 | 0.04 | 0.03 | 0.03 |
| FIN | - | - | - | 0.13 | 0.04 | 0.10 | 0.10 |
| AFR | - | - | - | - | 0.08 | 0.04 | 0.04 |
| NFE | - | - | - | - | - | 0.12 | 0.12 |
| ASJ | - | - | - | - | - | - | 0.12 |

Abbreviations: MAF, minor allele frequency; SNP, single nucleotide polymorphism; STMGP, Smooth-Threshold Multivariate Genetic Prediction; EAS, East Asian; AMR, Latino; FIN, Finnish; AFR, African/African American; NFE, Non-Finnish European; ASJ, Ashkenazi Jewish

The proportion of rare variants in the current Japanese datasets was 12%, and both rare and common variants contributed to the prediction of STMGP.

Supplementary Table 3

Partial correlations of STMGP with different τ parameters

|  | Partial correlations in independent validation datasets (SE) | Partial correlations in training datasets (SE) |
| --- | --- | --- |
| STMGP (τ=$N/\sqrt{log(N)}$) | 0.0530 (0.0180) | 0.3230 (0.0151) |
| STMGP (τ=$N/{0.1}$) | 0.0352 (0.0175) | 0.3739 (0.0150) |
| STMGP (τ=$N/1$) | 0.0689 (0.0172) | 0.3088 (0.0154) |
| STMGP (τ=$N/{10}$) | 0.0964 (0.0176) | 0.1690 (0.0159) |

Abbreviations: PCC, predictive correlation coefficient; SE, standard error; STMGP, Smooth-Threshold Multivariate Genetic Prediction.

Supplementary Table 4

Prediction accuracies based on the Box-Cox transformed phenotype and outlier-excluded datasets

|  | Box-Cox transformed | | Outlier-excluded | |
| --- | --- | --- | --- | --- |
|  | Partial correlations in independent validation datasets (SE) | Partial correlations in training datasets (SE) | Partial correlations in independent validation datasets (SE) | Partial correlations in training datasets (SE) |
| STMGP | 0.0577 (0.0175) | 0.2849 (0.0154) | 0.0435 (0.0183) | 0.2839 (0.0148) |
| PRS | 0.0040 (0.0180) | 0.6829 (0.0108) | 0.0075 (0.0192) | 0.5984 (0.0113) |
| GBLUP | 0.0163 (0.0184) | 0.9680 (0.0014) | 0.0086 (0.0181) | 0.9601 (0.0016) |
| SBLUP | 0.0033 (0.0184) | 0.9565 (0.0016) | 0.0081 (0.0182) | 0.9579 (0.0014) |
| BayesR | 0.0132 (0.0186) | 0.9567 (0.0016) | 0.0109 (0.0181) | 0.9589 (0.0014) |
| Ridge | 0.0162 (0.0180) | 0.9998 (0.0000) | 0.0051 (0.0187) | 0.9998 (0.0000) |

Abbreviations: PCC, predictive correlation coefficient; SE, standard error; STMGP, Smooth-Threshold Multivariate Genetic Prediction; PRS, polygenic risk scores; GBLUP, genomic best linear unbiased prediction; SBLUP, summary-data based best linear unbiased prediction.

Supplementary Table 5

Prediction accuracies with alternative methods for adjusting covariates

(Regressing out the covariates and using damage from GEJE)

|  | Covariates were included in prediction models.  Covariates: age, sex, significant PCs, and damage from GEJE | | Covariates were adjusted before building prediction models.  Covariates: age, sex, and significant PCs | | Covariates were adjusted before building prediction models.  Covariates: age, sex, significant PCs, and damage from GEJE | |
| --- | --- | --- | --- | --- | --- | --- |
|  | Partial correlations in independent validation datasets (SE) | Partial correlations in training datasets (SE) | PCCs in independent validation datasets (SE) | PCCs in training datasets (SE) | PCCs in independent validation datasets (SE) | PCCs in training datasets (SE) |
| STMGP | 0.0517 (0.0177) | 0.3784 (0.0148) | 0.0155 (0.0182) | 0.3928 (0.0153) | 0.0192 (0.0182) | 0.4638 (0.0145) |
| PRS | 0.0194 (0.0175) | 0.9324 (0.0076) | -0.0005 (0.0182) | 0.8076 (0.0089) | 0.0090 (0.0178) | 0.8835 (0.0081) |
| GBLUP | 0.0135 (0.0174) | 0.9628 (0.0017) | -0.0003 (0.0178) | 0.9610 (0.0018) | -0.0044 (0.0177) | 0.9604 (0.0018) |
| SBLUP | 0.0073 (0.0177) | 0.9539 (0.0019) | -0.0011 (0.0178) | 0.9564 (0.0018) | -0.0052 (0.0177) | 0.9562 (0.0018) |
| BayesR | 0.0121 (0.0183) | 0.9634 (0.0014) | 0.0080 (0.0185) | 0.9604 (0.0016) | 0.0117 (0.0187) | 0.9445 (0.0022) |
| Ridge | 0.0075 (0.0177) | 0.9998 (0.0000) | NA^a^ | NA^a^ | NA^a^ | NA^a^ |

Abbreviations: GEJE, Great East Japan Earthquake; PCC, predictive correlation coefficient; SE, standard error; STMGP, Smooth-Threshold Multivariate Genetic Prediction; PRS, polygenic risk scores; GBLUP, genomic best linear unbiased prediction; SBLUP, summary-data based best linear unbiased prediction.

^a^ Since the predicted phenotypes were the same among all the subjects, the PCC values could not be calculated.

Supplementary Table 6

Prediction accuracies with alternative methods for adjusting covariates

Predictive correlation coefficient without adjusting covariates

|  | PCCs in the independent validation datasets (SE) | *P*-value | PCCs in the training datasets (SE) |
| --- | --- | --- | --- |
| STMGP | 0.0769 (0.0173) | 2.114×10^-5^ | 0.3232 (0.0153) |
| PRS | 0.0322 (0.0176) | 0.06719 | 0.9127 (0.0076) |
| GBLUP | 0.0309 (0.0178) | 0.08806 | 0.9627 (0.0017) |
| SBLUP | 0.0192 (0.0178) | 0.3663 | 0.9554 (0.0019) |
| BayesR | 0.0211 (0.0185) | 0.5801 | 0.9633 (0.0015) |
| Ridge | 0.0260 (0.0178) | 0.1511 | 0.9998 (0.0000) |

Abbreviations: PCC, predictive correlation coefficient; SE, standardized error; STMGP, smooth-threshold multivariate genetic prediction; PRS, polygenic risk scores; GBLUP, genomic best linear unbiased prediction; SBLUP, summary-data-based best linear unbiased prediction.

The PCC of STMGP was significantly larger than that of the other models (*P*-values<0.05).

Supplementary Table 7.

Prediction accuracy based on imputed genome data

|  | Partial correlations in independent validation datasets (SE) | Partial correlations in training datasets (SE) | *P*-value for PCC | The number of variants included in prediction models | *P*-value cutoff for selecting SNPs |
| --- | --- | --- | --- | --- | --- |
| STMGP | 0.0628 (0.0173) | 0.1793 (0.0153) | 7.486×10^-4^ | 72 | 1.7×10^-5^ |
| PRS | 0.0015 (0.0176) | 0.8311 (0.0082) | 0.8570 | 9,005 | 1.5×10^-3^ |
| GBLUP | 0.0127 (0.0181) | 0.9521 (0.0022) | 0.2546 | 5,949,462 | NA |
| SBLUP | 0.0108 (0.0178) | 0.9535 (0.0013) | 0.2029 | 30,007^a^ | NA |
| BayesR | 0.0191 (0.0182) | 1.0000 (0.0000) | 0.1718 | 30,007^a^ | NA |
| Ridge | 0.0006 (0.0184) | 0.9998 (0.0000) | 0.6573 | 33,538^a^ | NA |

Abbreviations: PCC, predictive correlation coefficient; SE, standard error; CES-D, Center for Epidemiologic Studies-Depression Scale; SD, standard deviation; GEJE, Great East Japan Earthquake.

^a^ For SBLUP, BayesR, and ridge regression, the SNP data were clumped into approximately 30,000 SNPs based on a previous study ^11^ because of the substantial computational cost.

Reference

1. Cherlin S *et al*. Prediction of treatment response in rheumatoid arthritis patients using genome-wide SNP data. *Genet Epidemiol* 2018; **42**(8)**:** 754-771.
